# Statins in Non-alcoholic Steatohepatitis

**DOI:** 10.3389/fcvm.2021.777131

**Published:** 2021-11-24

**Authors:** Jose D. Torres-Peña, Laura Martín-Piedra, Francisco Fuentes-Jiménez

**Affiliations:** ^1^Lipids and Atherosclerosis Unit, Department of Internal Medicine, Maimonides Biomedical Research Institute of Cordoba (IMIBIC), Reina Sofia University Hospital, University of Córdoba, Córdoba, Spain; ^2^CIBER Fisiopatología de la Obesidad y Nutrición (CIBEROBN), Instituto de Salud Carlos III, Córdoba, Spain

**Keywords:** statins, NASH, cardiovascular disease, liver, aminotransferase

## Abstract

Non-alcoholic fatty liver disease (NAFLD) is the primary cause of chronic liver disease. The range is extensive, including hepatocellular carcinoma, cirrhosis, fibrosis, fatty liver, and non-alcoholic steatohepatitis (NASH). NASH is a condition related to obesity, overweight, metabolic syndrome, diabetes, and dyslipidemia. It is a dynamic condition that can regress to isolated steatosis or progress to fibrosis and cirrhosis. Statins exert anti-inflammatory, proapoptotic, and antifibrotic effects. It has been proposed that these drugs could have a relevant role in NASH. In this review, we provide an overview of current evidence, from mechanisms of statins involved in the modulation of NASH to human trials about the use of statins to treat or attenuate NASH.

## Introduction

Beyond cholesterol-lowering effects, statins exert anti-inflammatory, proapoptotic, and antifibrotic activities (1, 2) thus it's has been proposed that statins could have a relevant role in non-alcoholic steatohepatitis (NASH). In this review, we provide an overview of current evidence about the use of statins to treat or attenuate NASH.

## Physiopathology of Nash/Nafld

Non-alcoholic fatty liver disease (NAFLD) is a primary cause of chronic liver disease. The range is extensive, including hepatocellular carcinoma, cirrhosis, fibrosis, fatty liver, and NASH. Specifically, NASH can be classified as inflammation and steatosis without or with fibrosis (3) and it is well established that this condition is related to obesity, overweight, metabolic syndrome, diabetes, and dyslipidemia (4). At this point is important to note that NASH is a dynamic condition that can regress to isolated steatosis, remain at a constant level of activity, or progress to fibrosis and cirrhosis (3).

When signaling between hepatocyte, adipose tissue, and microbiome is not altered, the hepatocyte homeostasis is preserved. However, the presence of insulin resistance, disruption in lipid metabolism, inflammation, and oxidative stress results in disruption of the protective mechanisms of hepatocytes and finally these cells die. After the death of hepatocytes, a process for the repair of damage is initiated by the release of signals that activate immune cells, sinusoidal endothelium, stellate cells, and ductal cells that results in fibrogenesis and endothelial remodeling (5).

**Figure 1:**
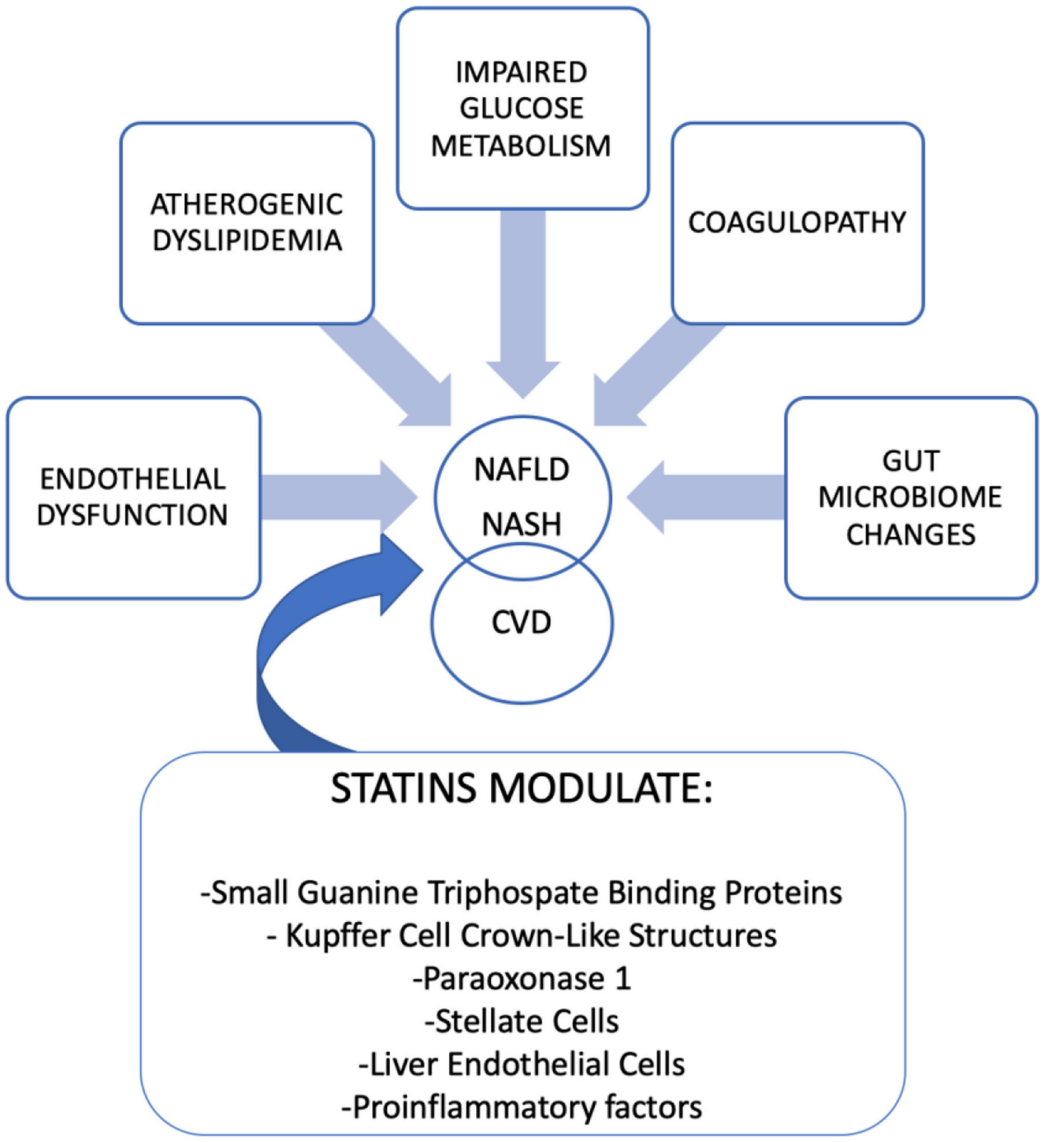
Physiopathology of NAFLD/NASH and link between Cardiovascular Disease (CVD). Mechanism of statins that modulate NAFLD/NASH.

Is important to note that inherited and environmental factors are involved in the physiopathology of NASH. On the one hand, genetic factors such as polymorphisms in patatin-like phospholipase domain–containing 3 (*PNPLA3*) and transmembrane 6 superfamily, member 2 (*TM6SF2*) promote the development of NASH. The first one is related to hepatic steatosis and cancer, and the second one modulates hepatocytes lipids content (6, 7). On the other hand, environmental factors may modulate NASH and evidence suggests that the gut-liver axis modifications are bidirectional: gut microbiota affects host obesity and hepatic diseases (including hepatic steatosis, NASH or cancer) and host factors (among others diet, sleep, shift-work, travel or feeding habit) influence the gut microbiota (8–10). An overview of the mechanisms involved in the pathogenesis of NASH that can be modified by statins is discussed below (see “Mechanisms of statins that can modulate NASH” and Figure 1).

## The Complex Link Between Nash/Nafld and Cardiovascular Disease

The mechanisms linking NASH/NAFLD to cardiovascular disease (CVD) are complex, varied, and include different pathways (Figure 1). As we previously mentioned, environmental factors, including sedentary lifestyle, diet rich in saturated fat, smoking, sleep disorders, the increase of visceral fat, and high body weight are key factors involved in NAFLD and NASH. Moreover, genetic variants of PNPLA3 and TM6SF2 are related to NASH and liver cancer but also to triglycerides, LDL cholesterols serum concentrations, and coronary heart disease (11). CVD is characterized by endothelial dysfunction and is typically associated with a decreased bioavailability of nitric oxide (NO). At this point, it is important to highlight that the presence of high levels of an antagonist of NO synthase, the asymmetric dimethylarginine (ADMA) is present in patients with NAFLD/NASH (12). In addition to endothelial dysfunction, a procoagulant state in these patients, characterized by elevated coagulation factor FXII to FVIII and fibrinogen, increases the risk of CVD (13). Alterations in glucose metabolism, diabetes, and hepatic insulin resistance are risk factors shared by NAFLD/NASH and atherosclerotic diseases (14). The imbalance in lipid metabolism that appears in NAFLD/NASH results in increased VLDL, IDL, LDL, triglycerides, and their remnants that develops atherogenic dyslipidemia (15).

## Pharmacotherapy in NASH/NAFLD

**Figure 2:**
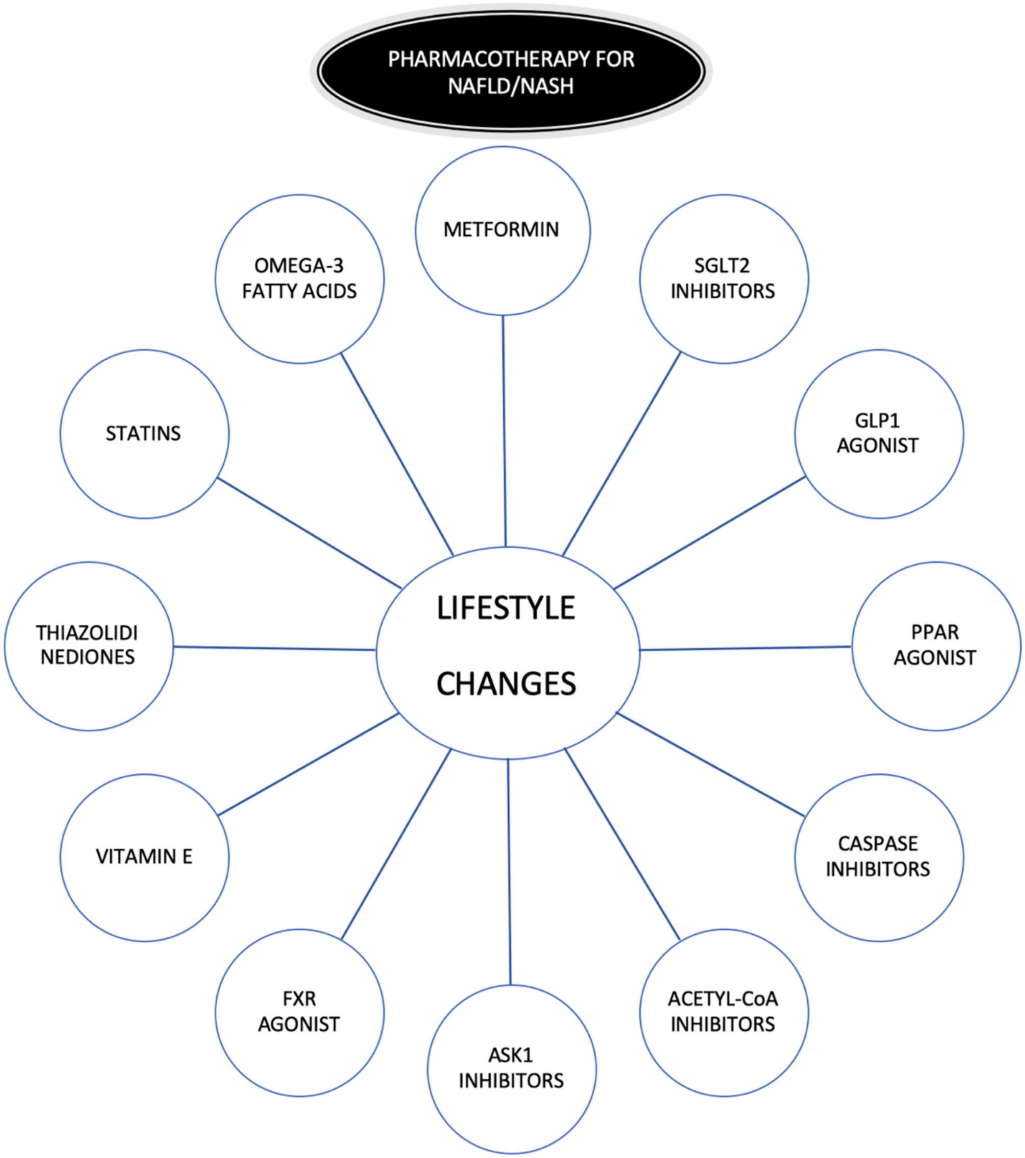
Pharmacotherapy in NAFLD/NASH. GLP1, Glucagon-Like Peptide 1; SGLT2, Sodium-Glucose Cotransporter-2; PPAR, Peroxisome proliferator-activated-receptor; ASK1, Apoptosis Signal-Regulating Kinase 1; FXR, inhibitors and Farnesoid X Receptor.

Lifestyle modifications and specifically weight loss, along with physical activity, is one of the best treatments (16), but lifestyle modifications are a challenge for patients and physicians because they are often difficult to maintain over time. This is the reason for the need to search for therapeutic targets to treat NASH/NAFLD (17) (Figure 2). The pharmacotherapies evaluated to treat NASH/NAFLD include “classic” therapies: metformin (18), thiazolidinediones (3), vitamin E(3), omega-3 fatty acids (19), and statins. Some of them only reported improvement in aminotransferase elevations (metformin and omega-3 fatty acids) and other improved histology (vitamin E, thiazolidinediones). The specific impact on aminotransferase and histology with the use of statins is discussed separately (see “the role of statins as therapeutic strategy in NASH/NAFLD patients”). On the other hand, there are novel therapies under investigation (20–23) alone or in combination (24, 25): Glucagon-Like Peptide 1(GLP1) Agonist, Sodium-Glucose Cotransporter-2 (SGLT2) Inhibitors, Peroxisome Proliferator-Activated-Receptor (PPAR) agonist, Caspase inhibitors, Acetyl-CoA inhibitors, Apoptosis Signal-Regulating Kinase 1 (ASK1) Inhibitors, and Farnesoid X Receptor (FXR) agonists. Some of these therapies increase serum lipids and the combination of statins with them will mitigate this effect (26). The results of phase 3 trials with these drugs will shed light on future treatment of NASH/NAFLD.

## Mechanisms of Statins that can Modulate NASH

### Small Guanine Triphosphate Binding Proteins (GTPases)

Statins may protect against NASH by inhibiting small GTPases. GTPases have diverse functions such as signal transduction, modulate protein synthesis, cell differentiation processes, or intracellular vesicle transport (27). In this line, a work developed by Schierwagen et al. (28) showed that treatment with simvastatin reduced liver inflammation and fibrosis in mice with NAFLD through inhibition of mice sarcoma protein. This work suggests that statins can change intracellular signaling. This mechanism may be responsible for the prospective liver effects in NAFLD/NASH.

### Statins and Proliferator-Activated Receptor (PPAR)

PPARs are receptors that bind fatty acids and regulate inflammatory and metabolic pathways. Specifically, PPAR α acts mainly in the hepatocyte modulating lipid transport, β-oxidation and gluconeogenesis genes (29). This PPARs receptor became an attractive target to treat NAFLD/NASH and clinical trials are ongoing to evaluate the effect of PPAR agonists in these diseases (21). Thus, the mitochondria and peroxisome have an important role in the degradation of fatty acids and in steatohepatitis by the accumulation of cholesterol: specifically, it has been suggested that mitochondrial and peroxisomal activity can be increased by statins, thus, a study conducted on mice (30) after the consumption of standard chow or a diet with a reduced content in choline and methionine without or with different statins improved hepatic fatty acid oxidation via PPARα and its target genes. Moreover, in this study, steatosis, inflammation, and fibrosis improved with the use of statins. Interestingly, these findings were not associated with the intensity of statins (atorvastatin, fluvastatin, rosuvastatin, pravastatin, and simvastatin). In summary, these findings are relevant: they suggest that statins treatment reduce hepatic steatohepatitis.

### Statins and Kupffer Cell (KC) Crown-Like Structures and Paraoxonase 1 (PON1)

PON 1 is an antioxidant enzyme from the liver and is related to a reduction in inflammation and antiatherogenic effects. The reduction of PON1 is a marker of lipid peroxidation that is improved by the use of statins (31). A trial conducted by Samy et al. (32) evaluated the use of statins in patients with NAFLD. 25 participants received 40 mg of atorvastatin daily for a period of 8 months and 25 received a placebo. The investigators evaluated, among others, liver ultrasonography, liver tests, lipids, and serum PON1 activity. After intervention, participants that received therapy with atorvastatin improved serum PON1 activity. KCs with crown-like structures and cholesterol are involved in inflammation, lipotoxicity, and fibrosis (33). In this context, a study in animal models (mice) investigator evaluated the effect of different interventions (34) including one group with ezetimibe, one group with atorvastatin, one group with atorvastatin plus ezetimibe, and one control group. Interestingly, animals that received atorvastatin and ezetimibe resolved cholesterol crystals, crown-like structures, and reduced fibrosis. In contrast, atorvastatin or ezetimibe alone developed a mild effect on these outcomes. This study provided evidence showing that the use of lipid-lowering drugs (including statins) reduces cholesterol crystals and modulate KC crown-like structures reducing NASH.

### Statins and Hepatic Stellate Cells Activation, Liver Endothelial Cell and Proinflammatory Factors

Hepatic stellate cells can be activated via paracrine of the hepatocyte and this activation results in fibrogenesis. This activation may be suppressed by statins treatment. Specifically, a study conducted using fluvastatin (35) in animal models showed that this treatment reduced profibrogenic pathways, showing the potential role of fluvastatin as an antifibrotic agent that could be used in NASH. Similar findings were demonstrated by simvastatin with *in vivo* and *in vitro* studies (1).

Liver endothelial cell function with reduced NO and stellate cells activation develop fibrosis. This could be modulated by statins. A study conducted in rats treated with simvastatin or atorvastatin (36) showed that statins reduced sinusoidal endothelial dysfunction, exerting the vasoprotective effects of these drugs.

Moreover, statins have anti-inflammatory and anti-fibrogenic effects. This was evaluated in a study that was developed to test the effect of rosuvastatin over different proinflammatory and profibrogenic factors (37). The rosuvastatin group showed a decrease in proinflammatory cytokines, including IL-6, TNF-α, IL-1β, and other angiogenic and profibrogenic factors. In this line, a trial with atorvastatin, vitamin E, and dietary intervention reduced the expression of fibrotic genes (38). All these findings support that statins could be protective against hepatic inflammation associated with NASH.

To sum up, statins improved liver endothelial cells dysfunction, receded activation of hepatic stellate cells, and exerted anti-fibrogenic effects.

## The Role of Statins As Therapeutic Strategy in NASH/NAFLD Patients

### Effects of Statins on Liver Histology

The etiology of NASH/NAFLD is influenced by inflammation and oxidative stress. Statins by their pleiotropic effects might improve liver histology. As previously mentioned, animal models showed how statins reduce the progression of fibrosis by antioxidant and antioxidant effects (1, 39).

Small uncontrolled investigations in humans used ultrasonography and tomography to assess the effects of statins on hepatic steatosis, and the majority found that they improved this condition. In this sense, it is important to highlight two studies: a randomized trial with atorvastatin in which this drug was more effective than fenofibrate in reducing liver echogenicity (40) and a randomized trial with atorvastatin (10–20 mg daily), where atorvastatin was shown to be as effective as pitavastatin in reducing steatosis (41). If we analyze data on the effect of statins on liver histology, the data are limited but despite this, the use of statins may be justified as an adjunct therapy to treat other conditions associated with NASH/NAFLD (prediabetes/type 2 diabetes) (42, 43). Some retrospective studies report improvement in steatosis without change in inflammation or fibrosis in patients treated with statins and interestingly, progressions in fibrosis in those that did not receive statins (44). In summary, we can conclude that most of the retrospective studies show how statins could reduce hepatic steatosis with improvement inflammation (45–47). The main limitation of these studies is the small number of patients included that may explain the different responses between participants.

## Long-Term Treatment: Safety and Efficacy in Patients With Altered Liver Tests

### Liver Toxicity of Statins. Use of Statins in Liver Diseases Including NASH

It is well established that elevations in serum aminotransferases are usual in patients that receive statins but in contrast (48), hepatic toxicity is not. In fact, the rate of acute liver failure in patients that are taking statins is very close to the general population (49). However, cases have been described of both autoimmune hepatitis associated with statin treatment (50) (Fluvastatin, atorvastatin, and rosuvastatin, among others) and acute liver failure requiring liver transplantation (0.01% of the causes of liver transplantation) (51). Another key point that may explain the alterations in liver function tests is due to the pharmacological interactions of statins rather than the toxic effect of these drugs (52).

Data from clinical trials such as the West of Scotland Coronary Prevention Study (WOSCOPS) (53), the Long-term Intervention with Pravastatin in Ischemic (LIPID) (54) study, and the Cholesterol And Recurrent Events (CARE) (55) suggest that long-term treatment with statins is tolerated without an excess of serious liver adverse events. On the other hand, meta-analyses have been developed to evaluate the effect of statins on liver function tests. One of them (56) analyzed almost 50,000 patients and showed that the use of statins (low or intermediate doses) was not associated with an alteration of liver enzymes. On the other hand, another study showed how comparatively high-dose statins increase the risk of transaminase elevation compared to low doses, especially in the case of hydrophilic statins (57).

Finally, it is important to highlight that the Statin Liver Safety Task Force established some years ago (58) the safety of statin treatment in patients with compensated cirrhosis, NASH, and NAFLD. In contrast, acute liver failure as long as decompensated cirrhosis contraindicate the use of statins.

### Treatment of NASH With Statins: From Animal Model to Human Clinical Studies

We have previously described in the first section of this manuscript many *in vitro*/*in vivo* studies evaluating the impact of statins in NASH through various mechanisms including their influence on pro-inflammatory factors, hepatic cell activation, sinusoidal endothelial cells, PON1, PPARα, and GTPases modulation.

There are no randomized controlled trials that evaluate the role of statins on NASH. We could only find data from *post hoc* analyses of prospective studies showing a beneficial effect of statins on NASH (Table 1).

**Table 1:** Summary of studies evaluating the effects of statin use in patients with NASH/NAFL.

**Authors (Ref)**	**Study design**	**Statin**	**Population health status**	**Sample size**	**Main findings**
Athyros et.al. (59)	Randomized trial (*post-hoc* analysis)	Atorvastatin vs. simvastatin	Patients with cardiovascular disease	437	-Participants treated with statins had improvement in liver tests (*p* < 0.0001) -Cardiovascular events occurred in 10% of patients with abnormal liver tests who received statin and 30% of patients with abnormal liver tests who did not receive statins (*p* < 0.0001)
Tikkanen et al. (60)	Randomized trial (*post-hoc* analysis)	Atorvastatin 80 mg/day vs. simvastatin 20–40mg/day	Coronary heart disease patients with normal and elevated baseline levels of serum alanine aminotransferase	1,081	Patients with elevated baseline aminotransferases, major cardiovascular event rates were 11.5% for simvastatin and 6.5% for atorvastatin, indicating a significant risk reduction with intensive statin therapy (HR, 0.556; 95% CI, 0.367–0.842; *p* = 0.0056)
Athyros et al. (40)	Randomized trial (*post-hoc* analysis)	Atorvastatin 30 mg/day vs. atorvastatin 20 mg/day.	Patients with metabolic syndrome without diabetes or cardiovascular disease	326	The number of events were none for the LDL-C <100 mg/dl group and 3 for the LDL-C <130 mg/dl group
NCT04679376 (active, not recruiting yet)	Randomized	Atorvastatin 40 mg/day vs. placebo	NASH patients	70	Primary objective: change in NASH score

*HR, hazard-ratio; CI, confidence intervals*.

In a subanalysis of the Greek Atorvastatin and Coronary Heart Disease Evaluation (GREACE) trial, which included coronary heart disease patients, the use of atorvastatin in patients with NASH/NAFLD improved liver enzymes and reduced cardiovascular events in those without alteration in liver tests after 3 years (59, 61).

The Assessing The Treatment Effect in Metabolic Syndrome Without Perceptible diabeTes (ATTEMPT) Study evaluated 1,123 participants with metabolic syndrome without diabetes or cardiovascular disease (60, 62). Participants were randomized into two groups: one with an LDL-C target under 130 mg/dl and the other with a target under 100 mg/dl. In the *post hoc* analysis liver enzymes and ultrasonography improved during the study.

Other relevant findings are those from a subanalysis from the Incremental Decrease in End Points Through Aggressive Lipid-Lowering (IDEAL) trial, which included >8,500 patients with established cardiovascular disease (60). Specifically, data from the *post hoc* analysis show that 1,081 have elevated ALT levels, probably due to NASH/ NAFLD. Participants with moderately elevated aminotransferase levels and received atorvastatin 80 mg daily normalized aminotransferase levels in contrast with those who received simvastatin 20–40 mg daily.

Other small trials, described in the previous section “Effects of Statins on Liver Histology” have been developed. These trials suggest the “biopsy-proven” effect of statins on NASH (40, 41, 44–47).

To our knowledge there is only one trial, with 70 participants (NCT04679376) (Table 1), to evaluate the safety and efficacy of statin therapy (atorvastatin 40 mg daily) for the treatment of NASH and hepatic fibrosis. This trial is active but not recruiting yet.

### Statins for the Treatment of Dyslipidemia in Patients With NASH

Dyslipidemia is a condition that is associated with NASH/NAFLD (42). Statins are the cornerstone of the treatment of hyperlipidemia. Although all statins seem to be effective to reduce cholesterol in patients with NASH, atorvastatin has a favorable profile to reduce the incidence of cardiovascular events in these patients. In the previous section we analyzed data from trials and effectiveness to treat NASH, but there is one important effect of these drugs in the participants involved in these trials: a reduction of CVD (Table 1). In the GREACE study, the subgroup of patients with NASH/ NAFLD that received statins had reduced CVD events, compared to those who had normal liver tests, and moreover, the investigators observed a reduction of CVD events compared with those with NAFLD without statins (59, 61). In the IDEAL study patients with a high dose of atorvastatin reduced the major CVD events (coronary and cerebrovascular events) compared to participants that received simvastatin (60). On the other hand, in the ATTEMPT study (62), the number of events were too low to establish a conclusion.

The latest ASL-EASD-EASO Clinical Practice Guidelines (63) support that the assessment of CVD risk must be developed in patients with NAFLD, and that they should receive treatment with statins if needed. Moreover, in the American Gastroenterological Association update for the treatment of Dyslipidemia in Common Liver Diseases (64), the author suggests that although NASH is not considered a traditional risk factor for the development of cardiovascular disease, it is commonly associated with hyperlipidemia, and statins are safe and well-tolerated in NASH patients to reduce CVD risk.

## Conclusions

Beyond cholesterol-lowering effects, statins exert anti-inflammatory, proapoptotic, and antifibrotic activities and could have a relevant role in NASH.

Many *in vivo* and *in vitro* studies evaluated the impact of statins in NASH through various mechanisms, including their influence on pro-inflammatory factors, hepatic cells activation, sinusoidal endothelial cells, crown-like structures, PON1, PPARα, and GTPases modulation.

Although there are no randomized controlled trials to evaluate the specific role of statins on NASH, there are *post hoc* analyses of prospective studies that demonstrate that statins have a positive effect on NASH. In this context, we need more clinical trials investigating the effect of statins on NASH/NAFLD and cardiovascular risk in these patients. However, at the current date, the guidelines support that the use of statins in patients with NAFLD/NASH is safe.

## Author Contributions

JT-P and FF-J: conceptualization and writing—original draft preparation. JT-P, FF-J, and LM-P: literature review and writing—review and editing. FF-J and LM-P: supervision. All authors have read and agreed to the published version of the manuscript.

## Conflict of Interest

The authors declare that the research was conducted in the absence of any commercial or financial relationships that could be construed as a potential conflict of interest.

## Publisher's Note

All claims expressed in this article are solely those of the authors and do not necessarily represent those of their affiliated organizations, or those of the publisher, the editors and the reviewers. Any product that may be evaluated in this article, or claim that may be made by its manufacturer, is not guaranteed or endorsed by the publisher.
